# Adapting a systematic conservation planning tool for supporting accessible and diverse urban greenspace recreation

**DOI:** 10.1007/s10980-026-02311-4

**Published:** 2026-02-21

**Authors:** Jieying Huang, Sarah E. Gergel, Melissa R. McHale

**Affiliations:** 1 Department of Forest & Conservation Sciences, University of British Columbia, 2424 Main Mall, Vancouver, BC V6T 1Z4 Canada; 2 Department of Forest Resources Management, University of British Columbia, 2424 Main Mall, Vancouver, BC V6T 1Z4 Canada

**Keywords:** Urban ecosystem services, Spatial prioritization, Greenspace accessibility, Urban greenspace equity, Urban landscape planning, Park amenities

## Abstract

**Context:**

Urban greenspaces are increasingly recognized for their multifunctionality—the capacity to provide diverse ecological and social benefits. Yet, planning strategies often focus on greenspace availability and accessibility, overlooking the functional and structural diversity within and among urban greenspaces. Traditional hotspot-based approaches typically prioritize areas with high richness, while overlooking rare and unique features that, despite their low abundance, may be critical to overall multifunctionality.

**Objective:**

With a focus on park amenities, this study explored the unique capabilities of Systematic Conservation Planning (SCP) for evaluating the diversity and complementarity of recreational opportunities in a region-wide urban park system. We further integrated mobility considerations to offer a more nuanced approach to greenspace monitoring. We asked: How do different measures of accessibility and mobility shape selection of park portfolios?

**Methods:**

As a proof-of-concept, we adapted a SCP approach, often used in biodiversity conservation, to identify greenspace portfolios in the City of Surrey, BC, Canada, a region with detailed mapping of diverse park amenities. Two contrasting scales of accessibility (neighborhood blocks vs. pixel-based) were used as constraints, and then evaluated under varying mobility (i.e. travel distance) assumptions.

**Results:**

We found finer scale (i.e. pixel-based) accessibility measures captured portfolios with a greater proportion of urban park amenities (80–100%) compared to block-level measures (1–67%), and also selected more spatially aggregated portfolios across the city. Irreplaceability patterns—indicating the parks most critical for diverse recreational amenities—varied depending on how accessibility was quantified. Lastly, more neighbourhoods were included in park portfolios as mobility (travel) distance increased, but this growth was non-linear.

**Conclusion:**

Our work demonstrates SCP’s potential as a valuable tool for planning and evaluating urban greenspace recreational opportunities. It offers a proof-of-concept for applying spatial prioritization in urban contexts, and can be feasibly expanded to include many additional aspects of park multi-functionality. Use of finer-scale measures of accessibility (as a cost constraint), and different mobility (i.e. travel distance) assumptions, further enhanced the selection of park portfolios that can provide diverse recreational opportunities.

**Supplementary Information:**

The online version contains supplementary material available at 10.1007/s10980-026-02311-4.

## Introduction

Urban greenspaces have gained increasing attention in urban planning and environmental management due to their “multifunctionality,” or the ability to simultaneously provide multiple functions (Hansen et al. 2017; Mitchell and Devisscher 2022). Urban greenspace provides a wide range of ecosystem services (ES) such as mitigating stormwater runoff (McPherson et al. 2011), improving air quality (Nowak et al. 2006), sequestrating carbon (Chen et al. 2014), and mitigating urban heat island effects (Mcpherson et al. 1997). Importantly, urban parks offer opportunities for outdoor recreation and provide benefits to physical and mental health of city residents (Konijnendijk et al. 2013; van den Bosch & Ode Sang 2017; Jarvis et al. 2020). Access to nearby greenspaces and parks for recreation and nature contact has been of great importance during the COVID-19 global pandemic (Venter et al. 2021). As urbanization continues, well-designed urban greenspace will play an important role in supporting the well-being of city residents around the world (Grima et al. 2020; Venter et al. 2021).

Most urban greenspace planning strategies focus on measures of greenspace “availability” and “accessibility” (Labib et al. 2020). Availability is often quantified by the existence of greenspace on a per-capita basis. For instance, cities in Germany aim to provide 6-10m^2^ of urban greenspace per person (Kabisch et al. 2016). In addition to availability, greenspace accessibility quantifies the proximity of greenspace to where people live using fixed distances and/or the shortest distance to greenspace (Labib et al. 2020). In planning, accessibility considerations aim to ensure residents have access to greenspaces within a certain distance (e.g., within 300 m of residential locations, as recommended by [WHO 2016]). The city of Berlin recommends access to a minimum of 0.5 ha urban greenspace within 500 m from home (Kabisch et al. 2016). Different accessibility measures have been linked to the health of city residents. For instance, access to nearby local parks (within 400 m) was associated with better health outcomes, such as lower obesity risk (Cutts et al. 2009), whereas greater distances to greenspace (beyond 1000 m) was associated with negative health outcomes (Pietilä et al. 2015).

Despite accessibility being among the most widely used indicators in urban greenspace research and planning (Labib et al. 2020), a lack of agreement on how to measure accessibility can contribute to inconsistent evaluation. Relying on proximity-based measures can obscure disparities across different urban areas and populations (Battiston and Schifanella 2024). For instance, the quality of greenspace, rather than distance, is a significant determinant of greenspace use (Schindler et al. 2022) and delivery of mental health benefits (Francis et al. 2012). Thus, there is a need to consider additional dimensions beyond accessibility for more effective greenspace planning.

Moreover, an individual’s mobility shapes their access to urban greenspace and any associated health benefits and recreational opportunities. Mobility capacity refers to the ability to physically reach greenspace and can vary with socio-demographic factors such as age, gender, and income level (Zheng et al. 2024). Mobility limitations can contribute to individuals, such as younger children and older adults, obtaining less greenspace recreational opportunities (Kabisch and Haase 2014; Battiston and Schifanella 2024). Mobility considerations are often overlooked in generalized proximity-based accessibility measures (Kabisch and Haase 2014; Battiston and Schifanella 2024). Thus, for demographics with limited mobility, considering both the availability and accessibility of neighborhood parks in a nuanced way is important. Unfortunately, incorporating mobility considerations into urban greenspace planning remains challenging, particularly in the design and location of park amenities (Ode Sang et al. 2016; Sundevall and Jansson 2020).

Furthermore, the diversity within and among urban greenspace is often overlooked in typical accessibility measures. Many such measures do not account for the diversity of potential recreation opportunities provisioned by different types of urban greenspace, nor preferences of different societal groups (Resler et al. 2023), nor potential overlap or gaps in amenities provided by a regional system of urban greenspaces. A range of recreational needs spans quiet parks and gardens for relaxation to more active areas like sports fields, playgrounds, and community gardens designed for physical activities and social engagement. In this sense, the presence and variety of amenities represent multiple recreational, cultural, and social ecosystem services, serving as a direct indicator of multifunctionality (Baró et al. 2017; Kimpton 2017; Hansen et al. 2019). Unlike greenspace metrics which are structural or vegetation-based, primarily reflecting ecological capacity, an approach focused on amenities reveal the realized and intentional functions of parks as spaces for diverse users, thus complementing ecological measures of multifunctionality (Gómez-Baggethun & Barton 2013; Haase et al. 2014). While multifunctionality can also arise from such ecological features and natural conditions, our focus here is on amenity-based recreational multifunctionality is useful because amenities provide a consistent, quantifiable, and activity-specific indicator of the types of recreation opportunities across a region, making the approach repeatable and easily understood for our “proof-of-concept” research addressed here. Lastly, addressing the diversity of park amenities may be particularly germane for larger parks (e.g., > 1 ha) which may offer a broader range of recreational amenities, such as sports, cultural activities, and nature exploration, compared to smaller parks with fewer basic amenities such as benches and small playgrounds (Li et al. 2024). Therefore, a singular focus on accessibility in urban greenspace planning can be further improved by incorporating the diversity of greenspace types and park amenities that cater to diverse recreational uses.

For all these reasons, planning that considers the multifunctionality of greenspace is of great need (Science for Environment Policy 2012). In considering multi-functional greenspaces, identification of hotspots is a common aim employing different weighting, prioritization, and multi-criteria decision analysis approaches (Cortinovis et al. 2021; Meerow and Newell 2017; Venter et al. 2021). However, these methods often focus on locations high in richness or abundance, evaluating hotspots as isolated units. As a result, such approaches may inadvertently overlook the value of rare and unique features which, despite being in low abundance, may play a critical role in supporting system-wide multi-functionality (Resler et al. 2023). By not considering how different locations may complement one another, such approaches risk missing an opportunity to create more balanced and representative regional networks of greenspace that can serve a greater diversity of societal needs.

In contrast, systematic conservation planning (SCP) is a complementarity-driven method that aims to include both high feature richness and track rare features, maintaining a balanced coverage of all desired features (Kukkala and Moilanen 2017). Instead of solely identifying individual locations of high richness or “hotspots”, SCP can identify sets (or combinations) of areas that jointly protect the most features. As such, SCP can help maintain a diversity of park features and avoid prioritizing two nearby greenspaces with similar park features or recreational opportunities for the same age group. Moreover, SCP identifies irreplaceable areas, those which are essential in maintaining overall regional diversity due to the presence of unique or rare features that might otherwise be overlooked.

In the past two decades, SCP has been used extensively to identify important areas for maintaining biodiversity (Amis et al. 2009; Snäll et al. 2016; Verhagen et al. 2018). SCP has also been applied within an ecosystem services framework to evaluate landscape multifunctionality and direct where management efforts should be implemented (Cimon-Morin et al. 2021). Few SCP examples address conservation goals in urban settings (Ettinger et al. 2021; Yu et al. 2023). This study addresses that gap by advancing the use of SCP beyond its traditional role in biodiversity conservation toward a novel application in urban greenspace planning. Our overarching goal is to demonstrate an innovative use of SCP, evaluating recreational opportunities in a region-wide system of parks, and furthermore, expand the use of SCP towards examining greenspace inequity as related to mobility considerations.

To advance this conversation and evaluate greenspace diversity for specific user groups, we adapt a systematic conservation planning (SCP) tool to identify greenspace portfolios based on their multifunctionality for recreation in the City of Surrey, BC, Canada. Specifically, we adapt SCP to evaluate the diversity and uniqueness of recreational amenities across a regional park system, thereby using the tool as a way to identify multifunctional greenspace portfolios that account for diverse recreational uses. To do so, we adapt SCP – with its typical emphasis on planning for species and ecosystems – to analyze recreational amenities of urban parks. We then use the approach to identify strategic combinations (or portfolios) of parks that provide diverse recreational opportunities for urban residents while at the same time considering accessibility constraints. We address the question: How do different measures of accessibility and mobility shape the selection of park portfolios? This approach can help decision-makers allocate management efforts towards park portfolios with the greatest diversity of amenities but also help identify areas that are “irreplaceable” in terms of their contribution to unique or distinctive amenities within a region.

### Methods

### Study area

Our study site includes the City of Surrey, a municipality of the Metro Vancouver Regional District (MVRD), which encompasses an exceptional system of urban parks along with particularly comprehensive mapping of park amenities. Surrey encompasses approximately 316 km^2^, with just over half a million residents (Statistics Canada 2021a). With land cover spanning agricultural uses to rapidly expanding urban uses, this fast-growing city is known as “The City of Parks” with over 400 parks within its boundaries (Surrey City Center, n.d.). Creating a variety of recreational spaces and activities for different age groups within a walkable distance remains a top priority in the city’s development plans (City of Surrey 2018). To guide these goals, the city has implemented various development plans and strategies (e.g., Walking Plan, Child- and Youth-Friendly City Strategy, and a Parks, Recreation and Cultural Strategic Plan) aimed at guiding the creation of walkable and inclusive greenspace to support the physical and mental health of residents. These local development goals align with the purpose of this research, making Surrey a good study site. Additionally, Surrey also offers open access to spatial datasets essential for this research.

### Park amenities

The spatial data for parks and amenities were downloaded from the city’s Open Data Portal (City of Surrey 2024) and then further rescaled for analysis purposes. The geodata collected by Surrey includes park boundaries (in polygon format) as well as park amenities (in polygon, line, and point forms) (see details in Table 1). We analyzed parks greater than one hectare in size, aiming to capture natural spaces large enough for recreation and physical activity (Annerstedt Van Den Bosch et al. 2016). Of the ten main groups of amenities originally identified in the dataset, playgrounds, recreation facilities, sports fields, and trails were further categorized into subgroups based on recreational interests of different age groups (See Table 1), for a total of 22 unique amenities. To account for the abundance of amenities within parks of different sizes, amenities were normalized by park size and examined as densities (number of amenities per ha).

**Table 1:** Summary of parks and associated amenities. Of the City of Surrey’s 864 total parks, we considered only those parks ≥ 1 ha and those with at least one type of amenity. In total, 271 parks were included in the analysis (median park size = 3.15 ha). Data summary at the block level is shown in online Table 4 (Appendix A). All spatial data were processed using ArcGIS Pro (version 3.3.1)

Categories	Total number of amenities	Number of parks with a given amenity	Rescaled amenity value (Mean per park)	Rescaling approach	Sources
Amenities (recreational features)					
Playgrounds*	165	94	0.013	The total area of each facility type within a park were rescaled by park size (ha/ha)	
Waterparks *	11	10	0.064		
Dog park*	20	20	0.287		
Fishing sites*	3	1	0.000004		
Garden*	11	11	0.032		
Trails (Paved)**	985	199	0.013	The total length of each trail within a park were rescaled by park size (m/ha)	
Trails (Unpaved)**	1288	209	0.012		
Picnic tables***	68	30	0.00003	The density was calculated for each facility (#/ha)	
Benches***	1454	10	0.00001		
Sports fields*					
Soccer fields	88	37	0.231	The total area of each facility type within a park were rescaled by park size (ha/ha)	Surrey Open Data Portal
Mini soccer fields	27	14	0.085		
Softball	55	25	0.097		
Basketball	31	27	0.013		
Other sport fields	45	20	0.118		
Recreation facilities*					
Volleyball	18	8	0.0057	The total area of each facility type within a park were rescaled by park size (ha/ha)	
Baseball	40	12	0.183		
Tennis court	71	18	0.012		
Pickleball	30	4	0.011		
Skate Park	8	6	0.017		
Bike Park	8	8	0.050		
Outdoor pools	7	7	0.006		
Other recreation facilities	61	11	0.017		

Spatial data in different formats: *polygon, ** line, ***point

For prioritization, we used local neighborhood blocks (formally named Dissemination Blocks) to define planning units representing the smallest geographic unit of analysis. In Canada, Dissemination Blocks (hereafter referred to simply as blocks) are the smallest geographic area over which census data are publicly-available (Statistics Canada 2021b). This fine scale of analysis works well with the high spatial resolution (5-m) mapping of greenspace, which will be introduced in the next section. ﻿Blocks were downloaded from the 2021 Canada Census website (Table 1). A total of 2501 blocks were used, omitting blocks that were split by city boundaries, or covered by water, or where park amenities data were not available.

To generate the spatial input for prioritization, each block was assigned a unique set of amenity values (using amenity abundances from its associated park). The details of each park amenity are described in Table 1. To account for size differences among amenities and parks, each park amenity was first rescaled by park size, with mean rescaled values in Table 1. This adjustment ensures that amenities such as benches, which vary in relative significance between large and small parks, are represented as density values rather than absolute counts, so their relative importance is comparable between large and small parks. By contrast, size-dependent amenities refer to features whose functional contribution depends on the proportion of park area they occupy rather than just their count. Examples include tennis courts, sports fields, or community gardens, which might occupy 1% of a large park versus 80% of a small park’s total area. Such amenities were therefore rescaled by proportional coverage within each park. For blocks encompassing more than one park, the abundance of all amenities from all of its parks was summed (and thus consolidated) into a single aggregated value (Online Table 4 in Appendix A). Finally, for each amenity, the aggregated value at the block level was normalized (to range between 0 and 1) to improve inter-comparability among amenities.

### Defining greenspace accessibility and mobility

In our approach, the distinction between accessibility and mobility is a nuanced but important one. Simply put, accessibility was used as a cost or constraint whereby portfolios with lower cost and better accessibility were emphasized. Thus, a dissemination block with a greater distance to greenspace would have a higher cost (and thus a reduced probability of selection). In this way, accessibility was conceptualized as the physical location of greenspaces. We further evaluated different mobility (i.e. travel distance) assumptions, which reflect that (for any given spatial arrangement of parks), some users will appreciate and realize different travel distances based on their mobility. These concepts and their implementation are explained in further detail below.

**Fig. 1:**
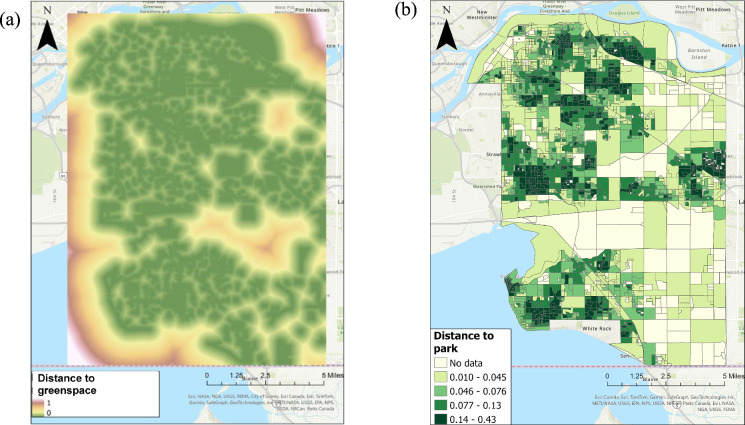
Contrasting examples of greenspace accessibility represented two ways: **a** at the scale of individual pixels and **b** at the scale of neighborhood (census dissemination) blocks. In both panels, the distance values were normalized between 0 and 1 km.Pixel-based accessibility (panel a) was calculated using a land cover dataset derived from high spatial resolution (5-m) imagery. Green in the legend indicates closer distances (more accessibility). Block-scale accessibility (panel b) was obtained from Canadian Urban Environmental Health Research Consortium (CANUE), representing the distance of a given block to a neighboring block with a park within a 1 km walking distance. In panel b, the values were visualized in five proximity categories with darker green indicates closer distances (more accessibility)

Furthermore, because of its fundamental importance, two contrasting approaches to quantifying accessibility were compared, each at a different spatial scale (pixel-level vs. block-level). Pixel-level accessibility was calculated using a land cover dataset of high (5-m) spatial resolution containing 12 urban classes with a high overall accuracy of 88% (Kappa 0.87) (Williams et al. 2019). Using this dataset, the Euclidean distance from each cell to the nearest greenspace cell was determined using ArcGIS Pro (version 3.3.1). Then, a unique cost value was assigned to each block (i.e., planning unit) using the median distance to greenspace within each block (Table 2). A visual example of accessibility and planning units is illustrated in Fig. 1.

**Table 2:** Summary of two types of costs (i.e. accessibility measures) used in Prioritizr. Costs are used as constraints in Prioritizer, and in the scenarios explored in this research, accessibility was used as the cost/constraint whereby areas with greater in distance to greenspace were assigned higher costs. Both pixel- and block-level accessibility measures were compared, generated from different sources

Costs	Defining accessibility	Data source
Accessibility – pixel-level	Euclidean distance from each cell within each dissemination block to the closest greenspace	Generated using high resolution land cover maps from William et al. (2019)
Accessibility – block-level	closeness of a dissemination block to any dissemination block with a neighborhood park within a 1 km walking distance	Canadian Urban Environmental Health Research Consortium (CANUE)

To better understand the impacts of spatial scale on prioritization output, another accessibility dataset was investigated. Obtained from CANUE (Canadian Urban Environmental Health Research Consortium 2021), it measures the distance of a given block to a neighboring block containing a park within a 1 km walking distance. To facilitate effective comparisons and understand similarities among subsequent portfolio outputs, Spearman’s rank correlation was used to assess the concordance between these two accessibility measures. ﻿

To examine how varying mobility assumptions may influence portfolio selection, we incorporated travel-distance-based scenarios. Three assumptions were evaluated, corresponding to the nearest 25, 50, and 75% travel distance thresholds. These distance thresholds are intended to loosely represent varying levels of mobility, as individuals with greater mobility—whether through physical ability, time, or access to transportation—can typically cover longer distances to reach parks. In this sense, mobility is conceptualized as the capacity to travel, and travel distance provides the measurable proxy (here, in km) by which it can be operationalized and compared. ﻿For example, a prioritization using the 25% thresholds yields park portfolio solutions that only considers blocks within the closet distance threshold; whereas the 75% thresholds would consider more parks with farther distance thresholds (Table 3). Rather than applying arbitrary fixed distances, this threshold-based approach provides a transparent, repeatable, and data-driven way to compare outcomes under different travel (i.e. mobility assumptions) distances, akin to a simplified sensitivity analysis.

**Table 3:** Travel distance (i.e. mobility) assumptions and thresholds used in the spatial prioritization analysis. Thresholds were applied using lockout constraints in prioritizr to represent 25%, 50%, and 75% of distance-to-park values

Travel distance thresholds definition	Interpretation	Constraint applied
25% nearest distance	Only the closest blocks considered; simulates limited access to park (e.g., children, elderly)	Lockout beyond 25%
50% nearest distance	Blocks within median distance; simulates moderate access to park	Lockout beyond 50%
75% nearest distance	Access to most parks within the region	Lockout beyond 75%

To implement these distance thresholds, the “add lockout constraints” function in Prioritizr package forced the solutions to only consider blocks within a specific accessibility threshold. While acknowledging this approach does not fully represent or capture all the nuances and dimensions of mobility, it does capture a simple and intuitive measure of a range distances people may be willing or able to travel. Indeed, empirical studies show that factors such as walkable proximity and independent travel distance play key roles in access to parks and opportunities (Rigolon 2016; Chen et al. 2024).

### Spatial prioritization with Prioritizr

We used the R package “Prioritizr” (Hanson et al. 2019) with Gurobi Optimization solver (Gurobi Optimization LLC 2024) to select a suite (or portfolio) of park amenities that are most accessible to residents. While a more technical description is outlined in Appendix B, here we describe the general flow. The input features included 22 rescaled park amenities (as described in Table 1), with dissemination blocks as the planning units, and two accessibility measures as planning unit costs. To guide the prioritization, we used the “maximum utility” objective function, which seeks to maximize the overall representation across the set of conservation features without exceeding a budget. In spatial prioritization, the budget often refers to the total amount of financial resources available for conservation efforts, setting the limit on what can be achieved. Similarly, cost refers to the financial expense associated with land purchases, management, and maintenance efforts. In our case, instead of using financial budget and cost, we used the accessibility of greenspace as the measure of cost and budget. Here, a maximum budget is defined as the sum of the maximum accessibility within each block (i.e., the sum of maximum costs across all blocks), which changes due to scale of accessibility. A more detailed workflow of our prioritization approach adapted to urban recreation is shown in Online Fig. 4 in Appendix B.

A park portfolio consists of a set of parks (planning units) that together maximize recreational diversity while reducing redundancies under defined accessibility constraints. Multiple park portfolios were generated from 100 iterations of the prioritization process. Portfolios were then evaluated and compared using two metrics which helped identify which particular blocks were repeatedly essential for building a strong portfolio. First, the selection frequency quantified how often each block was chosen across the 100 runs and is an indicator of which blocks were consistently included under varying scenarios of accessibility and amenities. Second, to quantify the concept of irreplaceability, an importance score was used reflecting its replacement cost. A replacement cost of zero indicates the block could be swapped in or out of a selected portfolio without affecting performance. In contrast, values closer to one express rising importance for a block in a final selected portfolio (Cabeza and Moilanen 2006).

## Results

### Characteristics of prioritization inputs

The following tables provide the summaries of input data for prioritization, including park amenities (Table 1), accessibility (Table 2), and travel distance (i.e. mobility) assumptions (Table 3).

### Accessibility comparisons

Accessibility was mapped in two different ways: at a pixel-level (Fig. 1a) and block-level (Fig. 1b). In general, the pixel-based approach resulted in a more widespread mapped distribution of accessibility throughout the study site and provided a continuous measure of accessibility. In contrast, for block-level accessibility, each block represents a unique proximity value. The darkest green represents the closest access to greenspace. Notably, there were substantial gaps in data (the lightest color) at the block-level, particularly from the city center to the east side. For the pixel-level measure, the median value of accessibility within each planning unit was used as the cost value for that planning unit. These cost values derived from each accessibility measure were subsequently used as inputs for further analysis.

### The impact of different accessibility measures on park portfolios

**Fig. 2:**
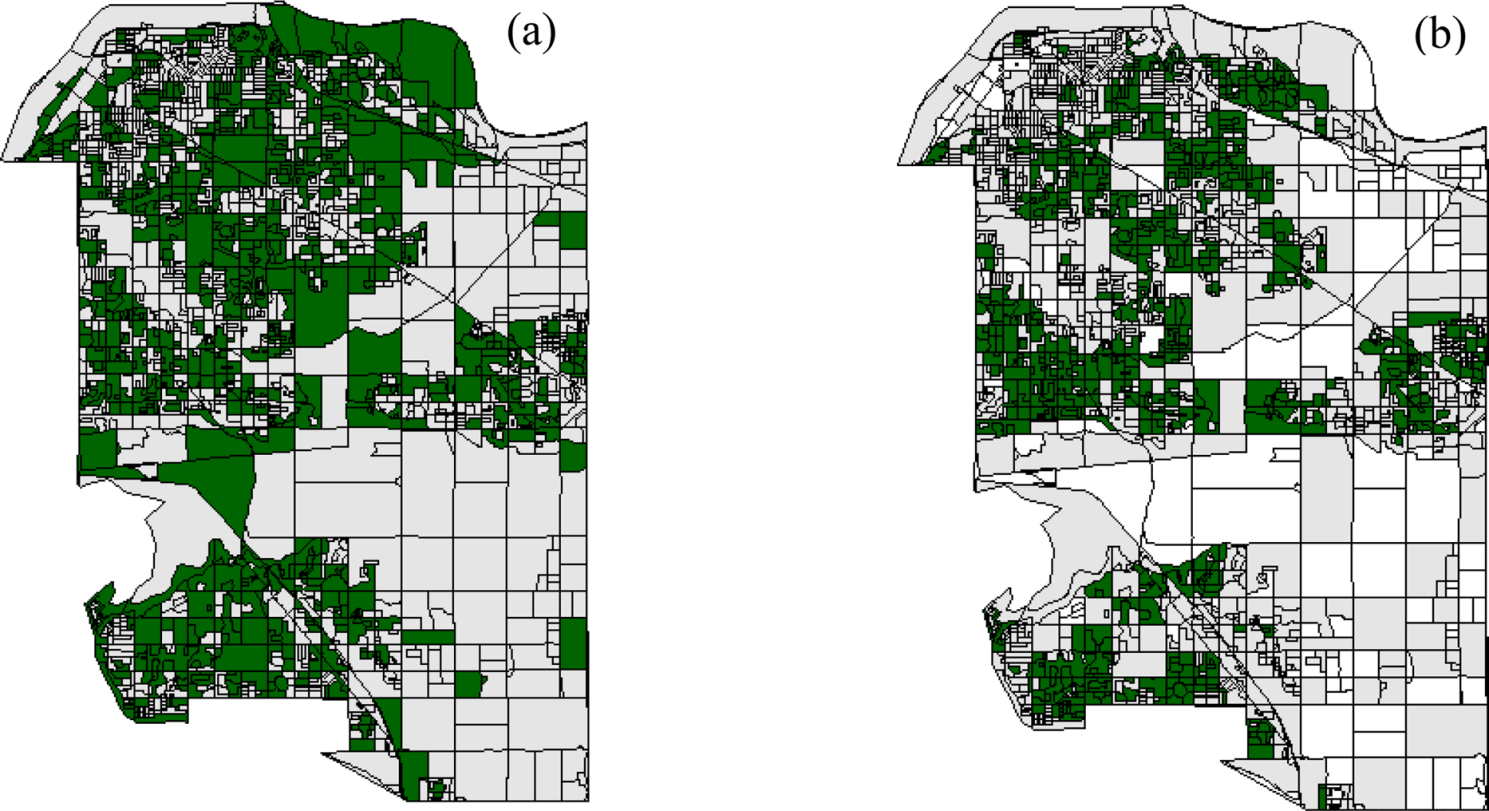
Selected park portfolios (i.e., selected dissemination blocks) for the City of Surrey using accessibility mapped at the pixel-level (Panel a) and block level (Panel b). Both used the median (50% threshold) travel distance (for the mobility assumption). Dark green indicates selected blocks, grey indicates unselected, and white indicates missing accessibility data

Different accessibility measures impacted the total amount and spatial patterns of selected park portfolios. Compared to the block-level, park portfolios selected using the pixel-based approach included more blocks. A total of 931 blocks were selected when using pixel-based accessibility, while only 727 were selected when using the block-level measure. When comparing the spatial patterns of park portfolios, the pixel-based approach resulted in greater aggregation of larger blocks across the city (Fig. 2a). In contrast, park portfolios using the block-level measure were more fragmented and dominated by small blocks (Fig. 2b).

Park portfolios using pixel-based measure captured 80% to 100% of park amenities, whereas those based on block-level measure only captured 1% to 67% of park amenities (Online Table 5 in Appendix A). In terms of specific amenities, soccer fields and basketball courts were among the top-represented, regardless of how accessibility was quantified. Importantly, portfolios using pixel-based accessibility were more effective at capturing scarce park amenities (Online Table 5 in Appendix A). For example, 90% of the rarest amenities (such as fishing sites, pickleball areas, and swimming pools) were captured when using pixel-based measure. In contrast, these rare amenities were hardly captured (< 13%) when using block-level accessibility.

### The impacts of differing travel distance (i.e. mobility) assumptions

**Fig. 3:**
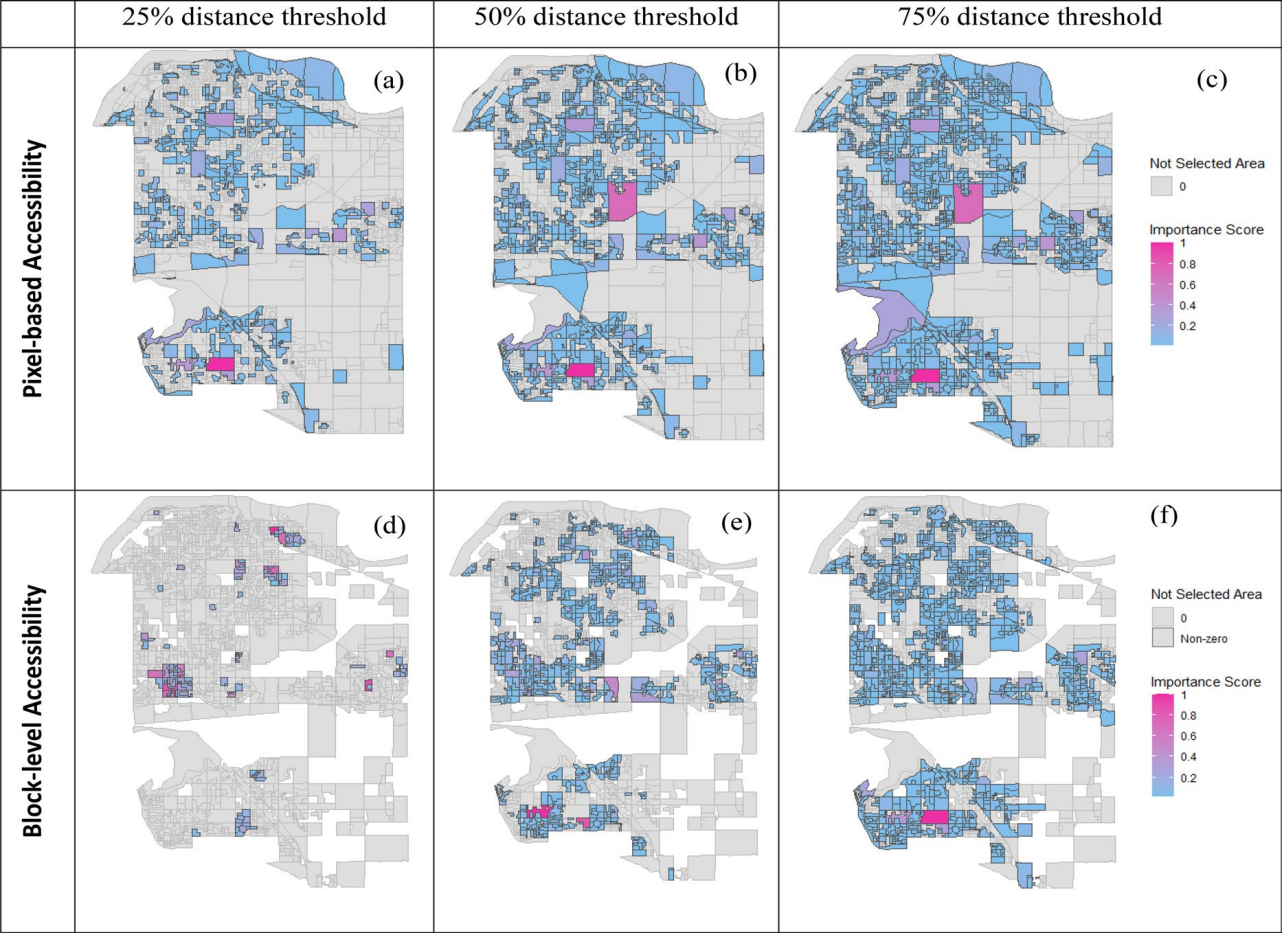
Spatial patterns and most irreplaceable blocks for park portfolios selected using different accessibility measures: at the pixel-level (top row) and block-level (bottom row). Three travel distances (i.e. mobility assumptions) are compared using the nearest 25%, 50%, 75% distance thresholds. The color gradient indicates the relative irreplaceability of selected blocks, with pink being the most irreplaceable (highest replacement cost) whereas blue indicates areas less costly to replace. Un-selected blocks are shown in gray

As the assumed travel distance increased, more neighborhood blocks were selected, regardless of the scale at which accessibility was quantified (Fig. 3); however, the increase was non-linear across travel-distance scenarios. The difference between 25 and 50% travel distance (443 blocks) was greater than the difference between 50 and 75% travel distance (422 blocks) using pixel-based accessibility. Similar patterns were observed for block-level measures. The spatial pattern of park portfolios became more aggregated as the travel distance increased, regardless of the way accessibility was quantified (Fig. 3).

In contrast, the number and location of irreplaceable areas captured in portfolios varied depending on the way accessibility was represented (Fig. 3). For pixel-based accessibility, the number of irreplaceable areas generally increased with farther travel distances. In contrast, using block-level accessibility, the number of irreplaceable blocks was highest at the nearest travel distance (25% threshold) and declined as travel distance increased (Fig. 3). The number of irreplaceable areas (those with importance scores > 0.6) increased with farther travel distances. However, the locations of the most highly irreplaceable blocks (those with importance scores nearing 1) remained consistent when using pixel-based accessibility. In contrast, when using block-level accessibility, the locations of the most irreplaceable areas shifted as travel distances changed, without any clear patterns observed.

Despite these variations, travel distance (mobility) assumptions did not impact the overall disparity in the representation of amenities between portfolios selected using pixel- and block-level accessibility (Table 5 in Appendix A). Especially for scarce amenities (such as fishing sites, pickleball areas, and swimming pools), increasing the travel distance threshold used for block-level accessibility only slightly increases the proportions (from 0 to 20%) of amenities captured. Similarly, increasing the travel distance threshold for pixel-based accessibility only slightly increases the proportions (from 80 to 100%) of rarest amenities captured.

## Discussion

### Spatial planning and the complementarity principle in an urban context

This paper introduces a novel approach to identifying important and accessible recreational urban greenspaces, with several unique contributions. Firstly, this study incorporates a spatial prioritization approach capable of collectively considering the diversity of park amenities across a large region in a way that provides a deeper consideration of multi-functionality. Conventionally, recreational opportunities in urban parks are often evaluated by scoring or ranking that focus on aggregated scores of amenities (Rigolon et al. 2018; Kraemer and Kabisch 2021). Such methods could prioritize two nearby parks with similarly high richness in amenities which serve identical user groups while overlooking rare amenities of interest to other user groups (Kimpton 2017). As highlighted by Kukkala and Moilanen (2012) and Margules and Pressey (2000), scoring and ranking approaches are not sufficient for robust prioritization because they ignore the principles of complementarity, representativeness, and irreplaceability. Compared to scoring and ranking methods, our approach offers a more nuanced understanding of urban park amenities by incorporating the principle of complementarity, which considers the joint function of several nearby parks in combination, ensuring that portfolios capture both common and rare features.

By applying spatial prioritization using complementary principles, our results for the City of Surrey identified parks with distinctive amenities such as gardens, swimming pools, and pickleball areas that would likely be overlooked by scoring-based methods. By accounting for complementarity, the analysis demonstrates how rare features contribute to overall recreational diversity across the region, thereby supporting a broader and more equitable view of multifunctionality in urban greenspaces (Jalkanen et al. 2020). This approach is particularly valuable in urban contexts where land is limited and planners must balance common amenities, such as playgrounds and sports fields, with rarer facilities that serve specific user groups. In doing so, complementarity-based prioritization provides a stronger basis for designing greenspace networks that reflect the diverse recreational needs of different user groups, ultimately improving our understanding of the multifunctionality of urban parks and their diverse park amenities across a city or a larger region. The approach is also highly adaptable and flexible enough to consider park attributes beyond park amenities.

### Improving monitoring of greenspace accessibility

Different spatial scales and resolution of data influence how patterns of accessibility, usage, and equity are characterized (Labib et al. 2020; Zhang and Tan 2023). Unlike previous studies that focus on greenspace accessibility (Dai 2011; Koppen et al. 2014; Texier et al. 2018), our research builds on these important contributions by offering two new perspectives on accessibility: mapping accessibility at two spatial scales (pixel- and block-level) as well as exploring the impact of different travel (i.e. mobility) distance assumptions. The scale at which accessibility was mapped (i.e., pixel- vs. block-level) impacted the selection of blocks in multiple dimensions, thus such decisions could potentially influence outcomes of greenspace planning more broadly. For instance, the block-scale accessibility (measured as per CANUE protocols) accounts for nearby greenspace within 1 km walking distance of a block; however, it overlooks greenspace within the block itself. In SCP/prioritization exercises, this limitation is further amplified with variable block sizes, particularly in larger blocks where internal greenspace is not accurately reflected in cost values, potentially leading to an underestimation of nearby recreational opportunities. More importantly, block-scale accessibility measures also ignored the arrangement of the parks, which can be more accurately assessed using fine-scale (e.g., pixel-level) accessibility measures.

Building on this scale comparison, we found that scenarios using finer scale (pixel-based) accessibility yielded more effective identification of unique or rare parks (as compared to using block-level accessibility). This finding aligns with other research pointing to the value of fine-scale assessments of urban greenspace quality, especially with regards to recreational opportunities (Derkzen et al. 2015; Tan and Samsudin 2017; Kraemer and Kabisch 2021; Wang et al. 2021). When analyzing specific park features, such as playgrounds, walking trails, or sports facilities, finer-scale assessments are particularly beneficial, as they offer a more detailed and accurate representation of the available recreational features and their usage patterns (Brown et al. 2014). Mapping accessibility with high-resolution data can support detailed mapping of park amenities which might otherwise be underestimated when using coarse accessibility measures. This level of detail is especially important when diversity in park amenities is a concern, as it enables identification of parks catering to a wide range of recreational needs. Others have also pointed to the need for high-resolution mapping for more effective urban greenspace planning (Reid et al. 2018; Labib et al. 2020). Taken together, these results suggest that the resolution of accessibility data not only influences how individual parks may be characterized but may also shape broader planning outcomes when identifying multifunctional greenspace portfolios.

In addition to the scale effects, our approach to examining differing travel distances (i.e. mobility assumptions) is practical, adaptable, and useful in examining equity in access. Firstly, we evaluated the concept of irreplaceability through an importance score. This approach reinforced how certain parks, even if not the largest or most feature-rich, could be essential in meeting the needs of populations with restricted travel. For example, a small greenspace with shaded seating near a senior community might emerge as highly irreplaceable (high importance score), especially when assuming a nearby shorter travel distance assumption. Our results showed that not only the total number of selected blocks but also the most irreplaceable blocks changed with assumptions made regarding travel distances. Logically, the number of blocks selected for a portfolio would increase under farther travel distance scenarios because more blocks, including those farther away, would be deemed suitable for inclusion. But particularly notable were highly irreplaceable blocks in the nearest travel distance portfolios, providing insights to parks with amenities that were not only rare but also more accessible. Previous work demonstrates that an individual’s travel distance positively supports their accessibility to greenspace and recreational opportunities (Koppen et al. 2014; Rigolon 2016; Wen et al. 2022; Lusseau and Baillie 2023).

### Future assessment of urban greenspace recreation

There are ways to refine our present spatial prioritization analysis. In this study, we focused on the density of amenities within each park as a proxy for one dimension of park quality. This amenity-based perspective reflects only one aspect of recreational multifunctionality. We recognize that multifunctionality also emerges from natural features, ecological conditions, and facility-based attributes not captured in our current analysis. Vegetation coverage, water bodies, park shape, and usage patterns (e.g., how often an amenity is used, and for how long), are all useful (Chan et al. 2018; Dade et al. 2020). Kraemer and Kabisch (2021) also developed a series of fine-scale park quality indicators such as tree diversity, stream density, distance to public transport, and traffic/emission exposure. Additionally, the built year of facilities and the frequency of maintenance also provide valuable insights to park condition and user experience. In future applications, incorporating these ecological and facility-based indicators—either as additional features or weighted layers—would offer a more holistic representation of recreational multifunctionality. In the Proritizr approach used here, such indicators could be added as additional feature layers, and/or weighting could be applied. Incorporating additional park quality and multi-functionality indicators could improve our current spatial prioritization analysis.

This spatial prioritization primarily considered the supply of parks and amenities, inadequately evaluating the role of residents’ demand (Wilkerson et al. 2018; Zhang and Tan 2023). Our representation serves as an approximation of supply (Tomscha et al. 2016) because it does not account for the location or density of residents. Park planning, which accounts for differences in population and demand, is important for enhancing the delivery of recreational services (Boone et al. 2009). Mapped population data, with individual layers for children, adults, and elderly people (representing the population size of each demographic group) could be used to more accurately reflect the potential beneficiaries of recreation opportunities and support age-specific planning.

Lastly, scale effects represent an important consideration for future assessments of urban greenspace recreation. As indicated in our results, both the number and location of the most irreplaceable areas were influenced by the scale at which accessibility was measured (pixel- vs. neighborhood block-scale). Broad-scale assessments can reveal general trends and patterns across extensive urban areas, while fine-scale assessments provide critical insights into localized phenomena that directly affect specific user groups. Future work should therefore move beyond single-scale analyses and systematically compare outcomes across multiple spatial resolutions. Such multi-scale assessments can reveal how planning results are sensitive to resolution choices and ensure that recreation opportunities are equitably represented at both neighborhood and regional levels. Incorporating multi-scale approaches into spatial prioritization would equip planners with more flexible and robust tools for identifying greenspace portfolios that reflect the diverse mobility capacities and recreational needs of urban residents.

### Caveats of current study

One of the main limitations of our study is its limited definition of greenspace. Other forms of green infrastructure, such as green roofs, bioswales, and rain gardens, play an increasingly important role in urban planning and provide valuable recreational opportunities. For instance, the Seattle Public Utilities Rain Wise program incorporates rain gardens that manage stormwater while also providing spaces for community enjoyment (The Seattle Times 2022). Moreover, urban recreational opportunities are not limited to greenspace. Bluespaces such as lakes, rivers, and wetlands provide unique recreational opportunities such as kayaking, bird watching, and educational tours. Although not explored here, our approach is flexible enough to incorporate any type of green- or bluespace which is adequately mapped. Including parks as well as additional forms of blue and greenspaces in prioritization would yield a more comprehensive perspective on urban recreational planning.

Handling the spatial complexities of urban features is challenging, especially when features overlap administrative boundaries. Thus, another caveat to this study was the need to aggregate and disaggregate spatial data for park amenities for block-level prioritization. Many parks did not correspond to a single block. Often, there were several parks within one block, or one large park spanning several blocks. By carefully reviewing the geodata layers, we ensured that amenities were neither lost nor double-counted, enabling a more accurate representation of urban parks. Such enhanced evaluation allowed us to better account for the spatial complexity of urban features and strengthen the prioritization approach.

Additionally, accessibility was estimated using Euclidean (straight-line) distances between dissemination blocks and their nearest greenspace. We acknowledge that this approach is a simplification, as it does not account for travel networks or barriers such as roads, buildings, or fences that influence real-world accessibility. Nevertheless, Euclidean distance remains a common and widely used proxy in greenspace accessibility studies because of its simplicity, transparency, and comparability (Comber et al. 2008; Neutens et al. 2010). For this proof-of-concept application, our use of Euclidean distance provides a valuable and reproducible baseline. Future research should incorporate more sophisticated approaches, such as network-based distances or travel-time analyses that incorporate road networks and travel modes (walking, cycling, transit), to provide more realistic measures of accessibility (Comber et al. 2008; Neutens et al. 2010).

In this study, mobility was represented using walking-based travel distance. However, in practice, individuals rely on a wider range of travel modes, including cycling, driving, and public transport, to access urban parks. These modes are also used unevenly across population groups—for example, cycling may be more common among adults, while children often depend on public transport or private vehicles. Future prioritization scenarios could therefore incorporate multiple travel modes by representing mobility using mode-specific travel distances or travel times (Jalkanen et al. 2020), which would improve the realism of mobility assumptions and strengthen equity considerations. Beyond travel mode, mobility and travel distance are also shaped by broader social factors. While our results indicated a general relationship between travel distances (i.e. mobility assumptions) and access to recreation, it is important to consider additional factors influencing travel distance, such as age (Wen et al. 2022), income level (Zheng et al. 2024), and transportation mode (Xu et al. 2017), all of which warrant further investigation. Children and the elderly are particularly affected by travel distance limitations which can restrict their ability to access greenspace (Kimpton 2017; Battiston and Schifanella 2024). Future research could further examine how access varies across age groups and socioeconomic contexts, supporting more equitable and inclusive urban greenspace planning (Rigolon 2016; Reuben et al. 2020; Sikorska et al. 2020). Exploring the factors influencing travel distance in greater detail would enhance urban planning efforts, leading to more equitable and inclusive recreational spaces.

## Conclusions

Our work is among the very few studies exploring the application of spatial prioritization, often used in biodiversity conservation, to identify urban greenspaces where differing mobility constraints and diverse recreational needs are incorporated. By exploring several measures of accessibility, we found that scenarios using finer scale (i.e., pixel-based) measures captured more urban park amenities than block-level accessibility measures. Moreover, changes in the number and location of parks with irreplaceable amenities were impacted by choices made for quantifying accessibility. Overall, our work demonstrated that SCP is a promising approach and well-suited for planning and evaluating a variety of nuances of urban recreational opportunities in urban greenspace.

## Supplementary Information

Below is the link to the electronic supplementary material.

Supplementary file1 (DOCX 70 KB)

## Data Availability

The datasets generated during and/or analyzed during the current study are available from the corresponding author on reasonable request
