# Supplementary material for: Adapting a systematic conservation planning tool for supporting accessible and diverse urban greenspace recreation
Source: Landsc Ecol. 2026 Feb 21;41(3):53. doi: 10.1007/s10980-026-02311-4 (PMC12979426; doi:10.1007/s10980-026-02311-4)
Supplement: Supplementary file 1 — Supplementary file1 (DOCX 70 KB) [file 10980_2026_2311_MOESM1_ESM.docx]

**Appendix A**

Table 4. Summary of dissemination block and associated amenity values. Of the original 2501 dissemination blocks (DBs) with median size of 4.1 ha, only 401 blocks contained 1 or more amenities and are summarized in the table below. For blocks encompassing more than one park, the abundance of all amenities from all of its parks were summed (and thus consolidated) into a single aggregated value, which then were rescaled between 0 and 1.

| **Categories** | **Total number of amenities** | **Number of DBs with a given amenity** | **Rescaled amenity value (Mean per DB)** |
| --- | --- | --- | --- |
| ***Amenities (or features)*** | |  |  |
| playgrounds* | 165 | 102 | 0.013 |
| Waterparks * | 11 | 11 | 0.390 |
| Dog park* | 20 | 20 | 0.272 |
| Fishing sites* | 3 | 1 | 0.000004 |
| Garden* | 11 | 11 | 0.030 |
| Trails Paved** | 985 | 249 | 0.014 |
| Trails Unpaved** | 1288 | 267 | 0.013 |
| Picnic tables*** | 68 | 38 | 0.00003 |
| Benches*** | 1454 | 17 | 0.000009 |
| ***Sports fields**** |  |  |  |
| Soccer fields | 88 | 46 | 0.231 |
| Mini soccer fields | 27 | 20 | 0.072 |
| Softball | 55 | 29 | 0.091 |
| Basketball | 31 | 31 | 0.011 |
| Other sport fields | 45 | 28 | 0.099 |
| ***Recreation facilities**** | |  |  |
| Volleyball | 18 | 14 | 0.004 |
| Baseball | 40 | 13 | 0.173 |
| Tennis court | 71 | 23 | 0.011 |
| Pickleball | 30 | 5 | 0.009 |
| Skate area | 8 | 7 | 0.016 |
| Bike area | 8 | 8 | 0.073 |
| Outdoor pools | 7 | 7 | 0.006 |
| Other recreation facilities | 61 | 12 | 0.016 |

Table 5. Summary of amenity representation (as a proportion of total occurrences of each amenity captured in a solution) in different park portfolios. Comparisons were made between two accessibility measures (pixel-level vs. block-levels). Three travel distances (i.e. mobility assumptions) including the nearest 25%, 50%, 75% distance thresholds were further tested for each of two types of the accessibility measure. The table reports results from the best solution, selected from 100 optimization runs for each scenario.

| **Amenities** | **Pixel-level Accessibility** | | | **Block-level Accessibility** | | |
| --- | --- | --- | --- | --- | --- | --- |
|  | **Travel distance thresholds** | | | | | |
|  | **<25%** | **<50%** | **<75%** | **<25%** | **<50%** | **<75%** |
| Playground | 0.676 | 0.962 | 0.999 | 0.217 | 0.490 | 0.778 |
| Waterpark | 0.699 | 0.998 | 1.000 | 0.239 | 0.417 | 0.548 |
| Picnic table | 0.816 | 0.986 | 0.999 | 0.256 | 0.541 | 0.686 |
| Bike park | 0.760 | 0.998 | 0.999 | 0.018 | 0.225 | 0.995 |
| Skate park | 0.991 | 1.000 | 1.000 | 0.108 | 0.432 | 0.891 |
| Soccer field | 0.910 | 0.997 | 0.999 | 0.403 | 0.693 | 0.917 |
| MiniSoccer field | 0.982 | 0.998 | 1.000 | 0.232 | 0.447 | 0.915 |
| Tennis court | 0.852 | 0.986 | 0.999 | 0.255 | 0.384 | 0.750 |
| Dog park | 0.708 | 0.998 | 0.999 | 0.079 | 0.619 | 0.788 |
| Basketball | 0.887 | 0.998 | 0.999 | 0.424 | 0.674 | 0.871 |
| Other recreation | 0.843 | 0.846 | 0.849 | 0.045 | 0.245 | 0.974 |
| Volleyball | 0.932 | 0.983 | 1.000 | 0.280 | 0.601 | 0.790 |
| Baseball | 0.995 | 0.999 | 1.000 | 0.206 | 0.563 | 0.999 |
| Softball | 0.895 | 0.995 | 1.000 | 0.252 | 0.477 | 0.725 |
| Other sportfield | 0.805 | 0.939 | 0.999 | 0.272 | 0.599 | 0.856 |
| Fishing site | 0.815 | 0.995 | 1.000 | 0.000 | 0.001 | 0.001 |
| Swimming pool | 1.000 | 1.000 | 1.000 | 0.000 | 0.123 | 0.176 |
| Bench | 0.867 | 0.999 | 1.000 | 0.354 | 0.542 | 0.700 |
| Pickleball | 0.982 | 0.991 | 1.000 | 0.000 | 0.033 | 0.219 |
| Garden | 0.940 | 0.985 | 1.000 | 0.044 | 0.163 | 0.999 |
| Unpaved trail | 0.673 | 0.805 | 0.945 | 0.119 | 0.355 | 0.578 |
| Paved trail | 0.702 | 0.962 | 0.996 | 0.251 | 0.524 | 0.744 |

Appendix B

**Spatial prioritization with Prioritizr**

1. *Conceptual framework*

Complementarity is a central principle in systematic conservation planning and guided the approach in this study for evaluating recreational diversity. In a conceptual sense, complementarity was assessed by the representation of unique and non-overlapping recreational amenities across selected planning units (parks), while reducing redundancy among amenities. In a mathematical sense, the prioritization solver treated each amenity type as a distinct feature, so it prioritized the portfolios that added new or underrepresented amenities rather than duplicating those already represented. This framing ensures that the optimization process favors combinations of parks that contribute the most novel amenities, which aligns with the broader principle of complementarity in systematic conservation planning. To operationalize this principle, we implemented it through a formal optimization framework.

The Prioritizr package uses Mixed Integer Linear Programming (MILP) to formulate and solve spatial prioritization problems in a systematic and mathematically precise way (Hanson et al., 2019). Unlike heuristic approaches such as simulated annealing, which approximate near optimal solutions, MILP solvers (e.g., Gurobi Optimization) provide an exact optimal solution by systematically evaluating all possible combinations of planning units within a set of predefined objectives and constraints. In this study, the objective was to identify a set of dissemination blocks that together offer the greatest diversity of park amenities, reducing redundancy while keeping accessibility-related costs within the budget. In practice, the prioritization steps involved: (i) defining dissemination blocks as planning units; (ii) setting each park amenity as a “conservation feature”; (iii) deriving several scenario-specific accessibility costs and assigning them to planning units; (iv) solving the optimization with the maximum-utility objective under budget constraints. The Gurobi Optimization solver evaluates all possible combinations using mathematical equations and logic rules to select the optimal solution (see relevant equations in the next section).

1. *Prioritization workflow*

**Conservation Features (Park Amenities in our case)**

**Cost (Accessibility at two scales in our case)**

**Portfolio: set of selected locations**

**Importance score for individual locations (i.e. irreplaceability)**


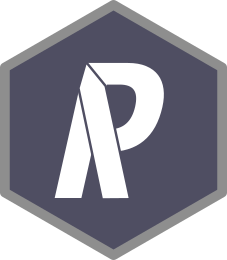


**Prioritizr Package in R**

Figure 4: Workflow of prioritization process. In general, Prioritizr helps create cost-effective spatial plans that meet predefined conservation goals while minimizing costs and working within constrains. Prioritizr requires users to convert the study area into smaller manageable units (i.e., planning units or PUs) that can be evaluated separately. The inputs for Prioritizr include conservation features and costs for each PU. In the context of this study, the conservation features are the park amenities, and the cost are represented by accessibility to nearby greenspace measured at two spatial scales. The outcomes of Prioritizr provide portfolios which identify optimal solutions (i.e., combinations of PUs that maximize the overall level of representation across all park amenities with the fewest units and minimum costs). It also reports the relative importance of individual PU (i.e., which are the most-irreplaceable blocks during the optimization process).

1. *Mathematical equations*

The Prioritizr package formulates spatial optimization problems using Mixed Integer Linear Programming (MILP) to identify the most efficient portfolio of planning units (in our case, dissemination blocks) that meets predefined representation targets for conservation features (in our case, park amenities), while minimizing costs (accessibility in our case). MILP allows for exact solutions by defining an objective function and a set of constraints, ensuring computational precision. In this study, the “maximum utility” objective function was used to maximize the overall representation of diversity across the set of park amenities without exceeding a predefined accessibility-based budget.

The “maximum utility” objective used in Prioritizr can be expressed mathematically for a set of planning units (𝐼 indexed by 𝑖) and a set of features (𝐽 indexed by 𝑗) as:

$$Maximize \sum_{i=1}^{I} -s c_{i}x_{i}+\sum_{j=1}^{J} a_{j}w_{j}$$

$$subject to$$

$$a_{j}= \sum_{i=1}^{I} x_{i}r_{ij}\forall j\in J$$

$$\sum_{i=1}^{I} x_{i}c_{i}\leq B$$

Here, $x_{i}$ is the decision variable (e.g., specifying whether planning unit 𝑖 has been selected (1) or not (0)), $r_{ij}$ is the amount of feature 𝑗 in planning unit 𝑖, $a_{j}$ is the amount of feature 𝑗 represented in the solution, and $w_{j}$ is the weight for feature 𝑗 (defaults to 1 for all features). The formulation inherently avoids redundancy by rewarding portfolios that add new or underrepresented features rather than duplicating those already captured. Additionally, 𝐵 is the budget allocated for the solution, $c_{i}$ is the cost of planning unit 𝑖, and 𝑠 is a scaling factor used to shrink the costs so that the problem will return a cheapest solution when there are multiple solutions that represent the same amount of all features within the budget.

The “maximum utility” objective function offers an efficient and transparent way to maximize the overall representation of features within a fixed budget, making it particularly suitable for large, complex systems (Csuti et al. 1997; Moilanen et al. 2009). The key advantage is computational simplicity: it ensures that as many features as possible are represented, often yielding high total utility scores with relatively low costs. However, a limitation is that it does not explicitly guarantee minimum representation targets for each feature, meaning that rare or unique features can be underrepresented if they contribute little to maximizing overall utility (Pressey et al. 1993; Margules & Pressey 2000). Nonetheless, because it rewards the inclusion of features not yet represented, the maximum utility approach still highlights the value of rare or unique features, especially when they contribute to the overall diversity of the portfolio. Thus, the maximum utility approach is valuable for proof-of-concept analyses or when broad coverage is the primary goal.
